# Change of tibial tuberosity-trochlear groove (TT-TG) distance during total knee arthroplasty had no influence on clinical outcome and anterior knee pain

**DOI:** 10.1007/s00264-021-05074-6

**Published:** 2021-06-01

**Authors:** Silvan Hess, Timo Fromm, Filippo Schiapparelli, Lukas B. Moser, Emma Robertson, Felix Amsler, Helmut Rasch, Michael T. Hirschmann

**Affiliations:** 1 Department of Orthopaedic Surgery and Traumatology, Kantonsspital Baselland (Bruderholz, Liestal, Laufen), CH-4101 Bruderholz, Switzerland; 2 University of Bern, Berne, Switzerland; 3 University of Basel, CH-4051 Basel, Switzerland; 4 Institute of Radiology and Nuclear Medicine, Kantonsspital Baselland (Bruderholz, Liestal, Laufen), CH-4101 Bruderholz, Switzerland; 5 Amsler Consulting, CH-4059 Basel, Switzerland

**Keywords:** TT-TG, Tibial tuberosity-trochlear groove distance, Total knee arthroplasty, Total knee replacement, Patellofemoral problems, Anterior knee pain

## Abstract

**Purpose:**

The main purpose of this study was to determine whether there is a correlation between the change of tibial tuberosity-trochlear groove (TT-TG) distance and clinical outcomes after total knee arthroplasty (TKA).

**Methods:**

A total of 52 knees undergoing TKA due to primary osteoarthritis were included in this retrospective study. All patients had pre- and postoperative CT scans. TT-TG distance was measured by two independent observers and the following alignment parameters were measured: hip-knee ankle angle (HKA), femoral mechanical angle (FMA), tibial mechanical angle (TMA), and posterior condylar angle (PCA). Clinical outcome was assessed using Knee Society Score (KSS) pre- and post-operatively and at a minimum of 12-month follow-up. Evidence of AKP was noted from follow-up reports. Pre- and postoperative scores were compared using a paired Student t-test. Pearson correlations were calculated to assess the influence of TT-TG on clinical outcome and of alignment parameters on the change in TT-TG. TT-TG between patients with and without AKP was compared using unpaired Student’s t-test (p < 0.05).

**Results:**

Neither the absolute postoperative TT-TG nor the amount of change in TT-TG correlated with the post-operative KSS or the change in KSS. Post-operative TT-TG and change in TT-TG did not differ significantly between patients with and patients without AKP. Only the change in FMA showed a correlation with the change in TT-TG (p = 0.01, r = 0.36).

**Conclusion:**

Despite a missing correlation between outcomes and TT-TG distance in this study, excessive TT-TG distance should be avoided. Furthermore, surgeons need to be aware that changes in femoral joint line orientation might affect TT-TG distance.

## Introduction

Patellofemoral problems are a common reason for dissatisfaction in total knee arthroplasty (TKA) patients. Up to 40% of all patients report anterior knee pain (AKP) after TKA and patellofemoral problems are among the most frequent reasons for early revision surgery [1–3]. The tibial tuberosity-trochlear groove (TT-TG) distance is a useful tool in the assessment of young patients with patellofemoral problems [4]. In these patients, a TT-TG distance greater 12 mm is considered “abnormal” and greater than 15 mm “pathological” [5]. To date, there is only a paucity of studies, which assessed TT-TG distances in osteoarthritic (OA) knees or patients undergoing total knee arthroplasty (TKA). Interestingly, these studies reported abnormal or even pathological TT-TG distance in a considerable number of OA patients [6, 7]. In addition, one study reported a strong correlation between TT-TG distances and overall lower limb alignment, whereby TT-TG distances increased with a more valgus limb alignment [7]. In a recent study, Nakamura et al. investigated the impact of postoperative TT-TG distances on clinical outcome as well as the relationship between TT-TG distances and component rotation, patellar tilt, and patellar shift [8]. Unfortunately, due to lack of pre-operative images, the authors where not able to assess the influence of TKA surgery on TT-TG distances nor the influence of change in TT-TG distances on clinical outcome.

The main purpose of this study was therefore to determine whether there is a correlation between the change in TT-TG distance and the clinical outcome after TKA. The secondary purpose was to assess if postoperative TT-TG distances greater than 12 mm or 15 mm are associated with more patellofemoral problems such as AKP. Finally, the third purpose was to assess which alignment parameters had an influence on the change in TT-TG distance during surgery. It was the primary hypothesis that the change in TT-TG distance from pre- to post-operatively correlated with clinical outcome (hypothesis 1a). Thereby, it was assumed that the greater the changes in TT-TG distance, the worse the clinical outcome would be (hypothesis 1b). The second hypothesis was that higher TT-TG distances post-operatively, regardless of the change, would result in worse clinical outcome. Thereby, it was specifically hypothesised that values greater than 12 mm or 15 mm will be associated with a worse clinical outcome (hypothesis 1c). The third hypothesis was that the change in femoral rotation and change in overall alignment would have an influence on the TT-TG distance (hypothesis 2).

## Material and methods

For this retrospective study, the hospital database was searched for patients meeting the following inclusion criteria: (i) received a TKA as treatment for primary knee osteoarthritis between 2009 and 2014, (ii) pre- and postop CT scans available according to the imperial knee protocol [9], (iii) available knee society scores pre-operatively and at minimum one year post-operatively. Patients were excluded if they had undergone corrective osteotomy of the femur or the tibia. A total of 52 knees (left: 23, right: 29) of 52 patients met the inclusion criteria (18 males and 34 females). Mean age ± standard deviation (SD) at time of surgery was 67.2 ± ten years (range: 43.6–67.2). The majority of patients (n = 34) received a cruciate retaining (CR) TKA whereas the others received a posterior stabilised (PS, n = 18) TKA. Patella was resurfaced in 13 patients.

**Fig. 1:**
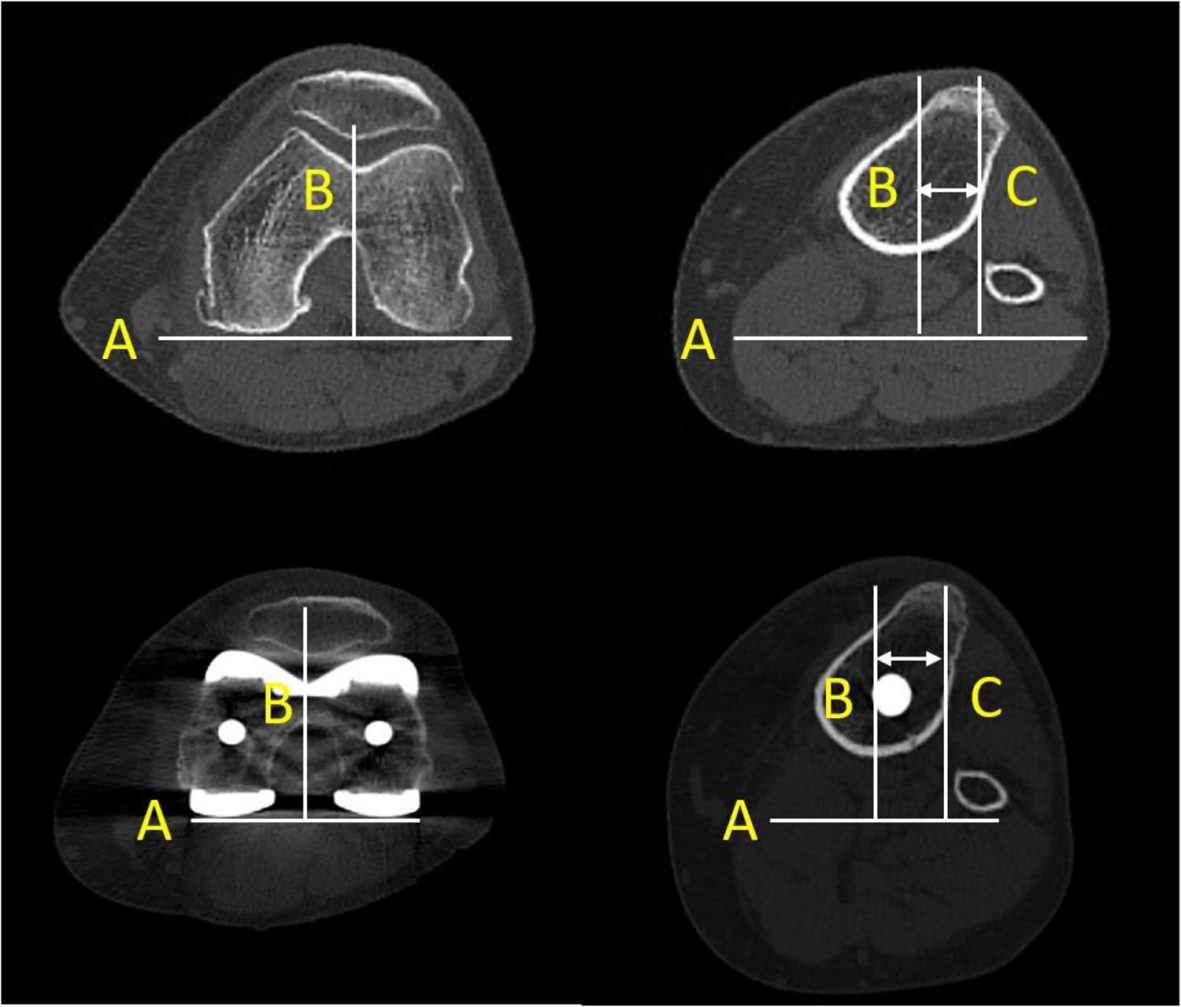
Measurements of tibial tuberosity-trochlear groove (TT-TG) distance pre-operative (above) and post-operative (below). First, the CT scan showing the deepest point in trochlea groove was selected and a tangent to the posterior condyles was drawn (line A). Then, a line perpendicular to line A was drawn through the deepest point of the groove (line B). These two lines were fixed (no change when selecting scrolling through scans), and the scan best showing the tuberosity tibiae was selected. Then, a second perpendicular line to line A was drawn through the most anterior point of the tuberosity tibia (line C). Finally, the distance between these two perpendicular lines was measured

TT-TG distance was measured according to the description of Schoettle et al. [10] as described in Fig. 1. The measurements were performed by two independent observers using Osirix Imaging Software (Pixmeo SARL, Geneva, Switzerland). Observer 1 was a novice with no previous experience and observer two was an experienced orthopaedic registrar. Both observers performed the measurements twice individually pre- and postoperatively with at least two week interval. Intra- and inter-observer reliability were tested with Cohen’s kappa coefficient and were found to be excellent according to the classification by Rosner et al. [11]. Pre- and post-operative intra-observer reliabilities were between 0.85 and 0.86. Pre- and post-operative inter-observer reliabilities were 0.92 and 0.93, respectively.

**Fig. 2:**
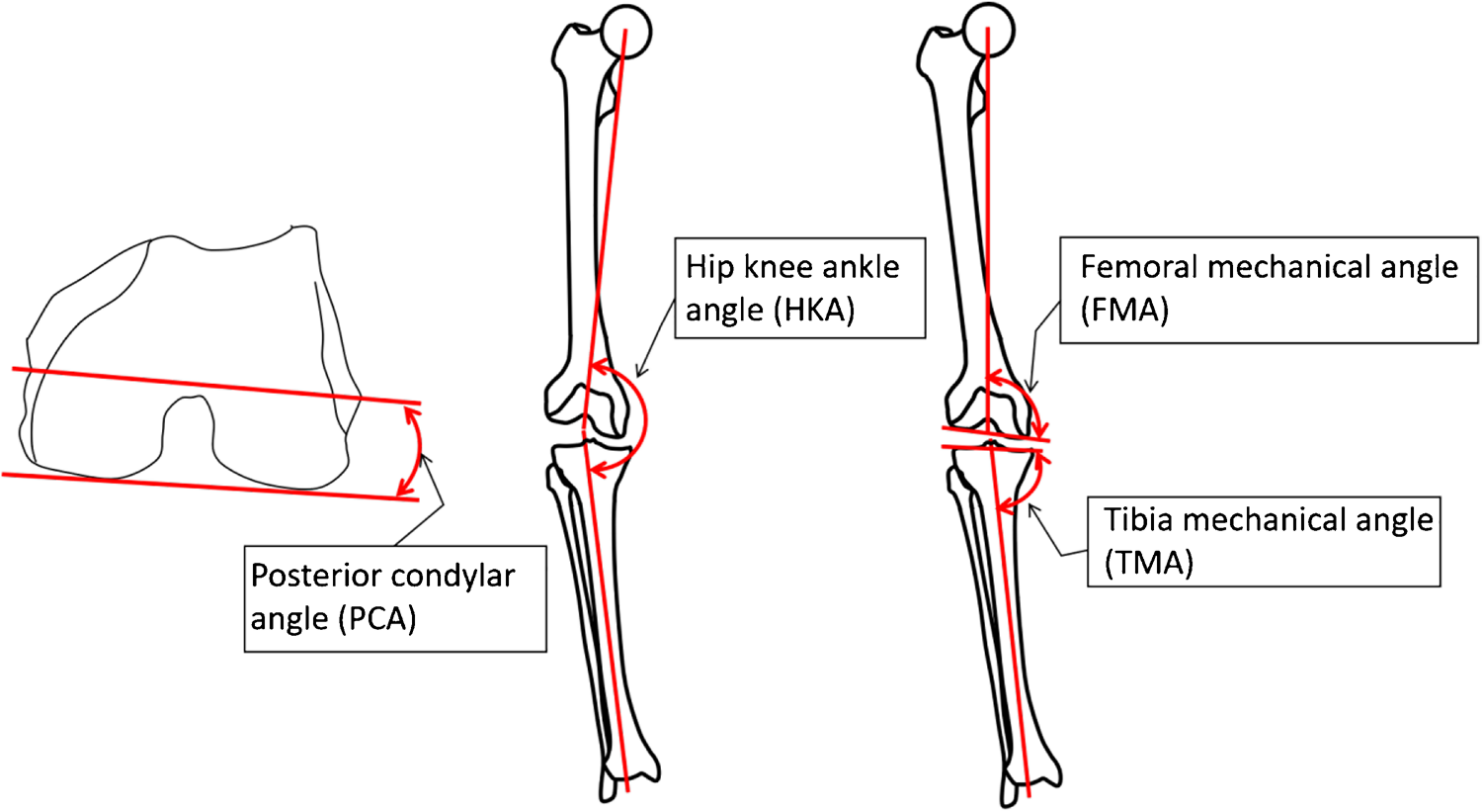
Measurements of alignment parameters. The hip-knee ankle angle (HKA) was defined as the angle formed by the lines connecting the centre of the femoral head to the knee and the talus to the knee. The femoral mechanical angle was defined as the angle between the femoral mechanical axis and a tangent to the distal femoral condyles. The tibial mechanical angle (TMA) was the defined as the angle between the tibial mechanical axis and a tangent to the proximal tibia joint surface. The posterior condylar angle (PCA) was defined as the angle between the transepicondylar line and a tangent to the posterior condyles

The following lower limb alignment parameters were measured using a customised analysis software (OrthoExpert©, London, UK) by an experienced observer: hip-knee ankle angle (HKA), femoral mechanical angle (FMA), tibial mechanical angle (TMA), and posterior condylar angle (PCA). Figure 2 shows the definition of these angles. FMA and TMA were both measured medially to be more coherent. By measuring them medially, a value above 90° will represent varus alignment of the femur and tibia, respectively.

The study was approved by the local ethical committee (EKNZ, 2016–02,221). All procedures performed were in accordance with the ethical standards of the institutional and/or national research committee and with the 1964 Declaration of Helsinki and its later amendments or comparable ethical standards.

## Statistical analysis

Descriptive statistics, such as means, ranges, and measures of variance (e.g. standard deviations, 95% confidence intervals (CI)) are presented. Pre- and post-operative values were compared using a paired Student t-test. Pearson correlations were calculated to assess the influence of TT-TG distance on clinical outcome and the influence of alignment parameter on the change in TT-TG distance during surgery. TT-TG distances between patients with AKP and without were compared using unpaired Student’s t-test. The level of statistical significance was p < 0.05.

Post hoc power analysis showed that, with the given N = 52, changes (2-sided) between pre and post-op values with an effect size of 0.40 (for example change of KSS (SD = 30) of 16 points) could be found with a power 80%. Pearson correlations > 0.37 could be proven with a power of 80%.

## Results

Table 1 shows the mean values ± standard deviations (SD) and ranges for all measured parameters. KSS, FMA, PCA, and TT-TG distances changed significantly from pre- to post-operatively (p < 0.001 for FMA, p < 0.01 for PCA, p < 0.001). Ten patients complained of AKP (19.2%) after TKA.

**Table 1:** Mean, standard deviation (SD), and ranges for pre-operative and post-operative values as well as amount of change in degrees or millimetres

	Pre-operative			Post-operative			Change pre- to post-op			
	Mean in °/mm	SD	Range	Mean in °/mm	SD	Range	p-Value	Mean in °/mm	SD	Range
HKA	178.96	6.36	170.0 to 194.0	179.65	2.69	174.0 to 185.0	0.351	0.7	5.3	− 15.0 to 10.0
FMA	92.37	3.04	85.0 to 98.0	89.81	2.38	85.0 to 95.0	p < 0.05	− 2.6	2.8	− 8.0 to 5.0
TMA	88.25	3.60	79.0 to 97.0	88.90	1.86	84.0 to 92.0	0.204	0.7	3.7	− 8.0 to 11.0
PCA	− 3.31	3.77	− 11.0 to 3.0	− 1.50	3.03	− 10.0 to 4.0	p < 0.05	1.8	4.9	− 9.0 to 11.0
TT-TG distance	1.15	0.38	0.32 to 1.97	0.55	0.48	− 0.45 to 1.68	p < 0.05	− 0.6	0.45	− 2.0 to 0.29
KSS	119.94	31.62	30.0 to 164.0	178.79	28.02	52.0 to 200.0	p < 0.05	58.85	38.62	− 57.0 to 160.0
KSS function	65.67	23.05	0 to 100.0	89.62	20.29	.0 to 100.0	p < 0.05	23.94	26.00	− 70.0 to 100.0
KSS knee	54.27	15.24	0 to 84.0	89.17	11.47	52.0 to 100.0	p < 0.05	34.90	19.19	− 7.0 to 100.0

*Hypothesis 1a and hypothesis 1b: Correlation between the change of TT-TG-distance and clinical outcome after TKA*

Neither the absolute post-operative TT-TG nor the amount of change in TT-TG correlated with the post-operative KSS or the change in KSS (pre- to post-operatively).

Post-operative TT-TG distance and change in TT-TG distance did not differ significantly between patients with AKP and patients without.

*Hypotheses 1c: Post-operative TT-TG distance greater than 15 mm or 20 mm is associated with more AKP*

One patient had a TT-TG distance greater 15 mm after TKA but did not report AKP.

*Hypothesis 2: Chang in femoral rotation and change in overall alignment influence the change in TT-TG distance*

Neither change in femoral rotation nor change in overall alignment correlated with the change in TT-TG distance. However, the change in FMA showed a significant correlation with the change in TT-TG (p = 0.01, r = 0.36).

## Discussion

The main finding of this study is that TT-TG distances did not have an influence on clinical outcome after TKA, and we thus must reject our first hypothesis (1a, 1b, 2c). TT-TG as a measurement for the lateralisation of the tibial tuberosity in relation to the trochlea femoris has been associated with elevated lateral patellofemoral contact pressure, increased lateral patellar tracking, and reduced patellar stability [12–14]. All these factors have been associated with patellofemoral problems in TKA patients such as AKP [2]. However, our results suggest that assessing TT-TG distances pre-operatively and proactively changing them during TKA might not be useful to reduce the rate of patellofemoral problems or improve outcomes after TKA. This is in accordance with a similar study by Nakamura et al. who assessed if postop TT-TG distances had an influence on the one-year post-op KSS in 115 consecutive patients with medial knee OA [8]. However, our study population differed distinctly from their study since we included all types of OA (e.g. medial, lateral, tri-compartment), used various types of implants and the patella was not resurfaced in all patients. More importantly, in the present study, the change in TT-TG was assessed in addition.

A second important finding of our study was that only the change in FMA correlated significantly with the change in TT-TG distance, and we therefore must reject our second hypothesis as well. Our results are in accordance with abovementioned study by Nakamura et al., who analysed the effect of TT-TG distance and component rotation on patellar tilt and patellar shift after TKA in 115 TKA patients using CT scans and axial radiographs [8]. They found significant correlations between post-operative TT-TG distance and post-operative patellar tilt and between post-operative TT-TG distance and post-operative patellar shift but not between post-operative femoral rotation and post-operative TT-TG distance. In contrast, Hochreiter et al. reported a positive correlation between the pre-operative overall coronal limb alignment and TT-TG distance [7]. They measured TT-TG distances of 962 consecutive patients (mean age ± SD 70.8 ± 9) from the KneePLAN 3D (Symbios Orthopédie S.A., Yverdon-Les-Bains, Switzerland) database and found that TT-TG distance increased 0.5 mm per degree increase in HKA. Consequently, it was argued that some patients with a varus alignment might be at risk for patellar malalignment after TKA, because their TT-TG distance would increase with surgery due to a valgisation of their overall alignment with the mechanical alignment concept. However, in our study, the change in HKA did not correlate with change in TT-TG and the mechanical TKA alignment concept leads to a decrease in TT-TG distances in nearly all patients (97.3%). This observation might be explained by the correlation between FMA and TT-TG. FMA is usually decreased when the mechanical alignment strategy is used (from valgus to neutral), and thus, TT-TG decreases as well.

The clinical consequences of our results are that they support the current practice of not routinely including TT-TG in the pre-operative planning for TKA. Furthermore, our results indicate that concerns regarding increasing TT-TG in varus knees when using the mechanical TKA alignment concept are unsubstantiated. Thus, in clinical practice, TT-TG should not be seen as an indication for any additional procedure such a patella resurfacing or a lateral release. It remains to be seen if post-operative pathological TT-TG distances will be associated with patellofemoral problems. Finally, based on our findings, surgeons need to be aware that in patients with a varus-aligned femur, a valgisation of the femur might lead to increase in TT-TG distances.

Several limitations need to be acknowledged. The lack of correlation between TT-TG postoperative distance or change in TT-TG distance and clinical outcome may be explained by several limitations of our study. Firstly, the outcome measurement tool (KSS) used is not specific to patellofemoral problems. To overcome this limitation, patient’s letters were screened for AKP. Secondly, our patient sample included patients with and without primary patellar resurfacing as well as patients in which a medial or lateral approach was used. Further limitations are the use of two different prosthesis designs (CR, PS) and prostheses from different manufactures. Although the prosthesis designs should not have influenced TT-TG distance, it could potentially have influenced clinical outcome. Our simple size was relatively small, and our post hoc power analysis indicates only a sufficient power to detect correlation and differences of moderated effect size. Our study should thus have been able to detect clinically meaningful differences but further studies, including more patients, are necessary to confirm our findings.

## Conclusion

Despite a missing correlation between outcome and TT-TG distance in this study, excessive TT-TG distance should be avoided. Furthermore, surgeons need to be aware that changes in femoral joint line orientation might affect TT-TG distance.

## Data Availability

Request for access to anonymised data and statistical analysis can be addressed to the corresponding author. Access will be granted on an ad hoc basis, depending on request/proposed usage. The data that support the findings of this study are available from the corresponding author, MH, upon reasonable request.

## Code availability

Osirix Imaging Software (Pixmeo SARL, Geneva, Switzerland); Analysis software (OrthoExpert©, London, UK).
